# Differential Protein Expression in Honeybee (*Apis mellifera L.*) Larvae: Underlying Caste Differentiation

**DOI:** 10.1371/journal.pone.0013455

**Published:** 2010-10-20

**Authors:** Jianke Li, Jing Wu, Desalegn Begna Rundassa, Feifei Song, Aijuan Zheng, Yu Fang

**Affiliations:** Key Laboratory of Pollinating Insect Biology, Department of Beekeeping and Biotechnology, Ministry of Agriculture, Institute of Apicultural Research, Chinese Academy of Agricultural Science, Beijing, China; Institut Européen de Chimie et Biologie, France

## Abstract

Honeybee (*Apis mellifera*) exhibits divisions in both morphology and reproduction. The queen is larger in size and fully developed sexually, while the worker bees are smaller in size and nearly infertile. To better understand the specific time and underlying molecular mechanisms of caste differentiation, the proteomic profiles of larvae intended to grow into queen and worker castes were compared at 72 and 120 hours using two dimensional electrophoresis (2-DE), network, enrichment and quantitative PCR analysis. There were significant differences in protein expression between the two larvae castes at 72 and 120 hours, suggesting the queen and the worker larvae have already decided their fate before 72 hours. Specifically, at 72 hours, queen intended larvae over-expressed transketolase, aldehyde reductase, and enolase proteins which are involved in carbohydrate metabolism and energy production, imaginal disc growth factor 4 which is a developmental related protein, long-chain-fatty-acid CoA ligase and proteasome subunit alpha type 5 which metabolize fatty and amino acids, while worker intended larvae over-expressed ATP synthase beta subunit, aldehyde dehydrogenase, thioredoxin peroxidase 1 and peroxiredoxin 2540, lethal (2) 37 and 14-3-3 protein epsilon, fatty acid binding protein, and translational controlled tumor protein. This differential protein expression between the two caste intended larvae was more pronounced at 120 hours, with particular significant differences in proteins associated with carbohydrate metabolism and energy production. Functional enrichment analysis suggests that carbohydrate metabolism and energy production and anti-oxidation proteins play major roles in the formation of caste divergence. The constructed network and validated gene expression identified target proteins for further functional study. This new finding is in contrast to the existing notion that 72 hour old larvae has bipotential and can develop into either queen or worker based on epigenetics and can help us to gain new insight into the time of departure as well as caste trajectory influencing elements at the molecular level.

## Introduction

Reproductive division of labor is characteristic of social insects, in which only some of the members are fully endowed with reproductive capacity, while others are not. As one of the highly developed social insects, the honeybee (*Apis mellifera* L.) also exhibits reproductive division of labor. This trait is not determined by genetic difference, but rather by complex sequential events occurring at early larval development stages. This complex sequence of events determines the fate of young and genetically identical larvae to switch into either reproductive a queen or infertile worker honeybee castes. According to the literature, larvae that are raised as queens or workers vary to a great extent in their gene and metabolic enzyme expression patterns, which are directly linked to caste determination [1]–[5]. A number of studies have been conducted on the differences between honeybee workers and queens, mainly focusing on the core causes of caste divergences. For instance, physiological and anatomical differences between the two castes [6]–[10], environmental or early larval feed factors [5], [11]–[14], differential gene expression[1], [2], [15], influence of juvenile hormone on caste determination [13], [16]–[18], and comparisons of hemolymph protein composition[19] have been reported. There is an existing notion that 3 day old larva is bipotent and can grow into either queen or worker depending on epigenetics. Therefore, our hypothesis is that there will be very slight differences in protein profiles of day 3 larvae from the queen and the worker. On the other hand, despite the aforementioned studies on the mechanisms of honeybee caste differentiation, little is known about the roles of primary proteins expressed at the early post embryonic stage. Therefore, the aim of this study is to investigate the specific time and the influences of primary proteins on post embryonic caste determinations in honeybee by using proteomic analysis in combination with bioinformatics.

## Materials and Methods

### Chemical Regents

Urea, Tris-base, sodium dodecyl sulfate (SDS), sodium bicarbonate (NH_4_HCO_3_), dithiothreitol (DTT), iodoacetamide and bovine serum albumin (BSA) were purchased from Sigma (St. Louis, MO, USA). Bio-lyte was obtained from Bio-Rad Laborataries (Hercules, CA, USA). Acrylamide, N,N'-methylenebisacrylamide, Ammonium Persulfate (AP), N,N,N',N'-tetramethylethylene diamine (TEMED), 3-[(3-cholamidopropyl)-dimethylammonio]-1-propane sulfonate (CHAPS), glycerol, Bromophenol Blue, Coomassie Brilliant Blue (CBB) G-250 α-cyano-4-hydroxycinnamic acid (CHCA) were manufactured by Bruker Daltonics (Billerica, Mass. USA). Trypsin (Modified, Sequencing Grade) was purchased, from Roche (Roche, Mannheim, Germany), and Trifluoroacetic acid (TFA) and acetonitrile were obtained from J. T. Baker (Phillipsburg, NJ, USA). Other chemicals used but not specified here were noted with their sources in the text.

### Biological Samples

The larvae of worker and queen honeybees (*Apis mellifera* L.) were randomly collected at 72 (3 days), 120 hours (5 days) from a frame in May 2009 at the Institute of Apicultural Research, Chinese Academy of Agricultural Science. To guarantee that the exact aged larvae were sampled, the queen was confined to a single wax comb frame containing worker cells for 5 hours with a cage made of a queen excluder, through which workers but not the queen could pass. Subsequently, the queen was removed and the eggs contained in the frame were maintained in the honeybee colony for further development. After the eggs had hatched, the young larvae were transferred from the worker cells to the queen cell cups in a queen rearing frame and put into the queenless colony to sample queen larva. The worker larvae were collected directly from the worker cells. A total of 100 larvae were sampled for the queen and the worker larvae at each time point and the homogenates were frozen at −80°C until use, and an aliquot of 10 g homogenate larvae were used for protein extractions.

### Protein Extraction and Two-dimensional Gel Electrophoresis (2-DE)

Larval protein extractions were carried out according to our previously described method [20]. Protein concentration was determined according to the Bradford method. BSA was used as standard and the absorption was measured at 595 nm (Beckman, spectrophotometer DU800). The extracted protein (380 µg from each sample with 3 replicates) were resuspended in lysis buffer (LB) [8M urea, 2M thiourea, 4% CHAPS, 20 mM Tris-base, 30 mM DTT, 2% Bio-lyte pH 3–10] and then mixed with rehydration buffer [8M urea, 2% CHAPS, 0.001% bromophenol blue, 45 mM DTT, 0.2% Bio-lyte pH 3–10]. The mixture was loaded onto a 17 cm IPG strip (immobilized pH gradient, pH 3–10, linear, Bio-Rad). Isoelectric focusing (IEF) was performed at 18°C according to manufacturer's instructions (Protean IEF Cell, Bio-Rad). Before sodium dodecyl sulfate polyacrylamide gel electrophoresis (SDS-PAGE), the IPG strips were first equilibrated for 15 minutes in equilibration buffer 1 [6M urea, 0.375M Tris-HCl (pH 8.8), 20% glycerol, 2% SDS, 2% DTT] followed by equilibration in buffer 2 [6M urea, 0.375M Tris-HCl (pH 8.8), 20% glycerol, 2% SDS, 2.5% iodoacetoamide] for another 15 minutes. Then, the strips were transferred onto 12% SDS polyacrylamide gel (1.00 mm). Second dimension electrophoresis, SDS-PAGE, was performed on a Protean II Xi Cell (Bio-Rad) at 25 mA/gel for 6 hours.

### Image Acquisition and Statistics Analysis

Gels were fixed overnight in 50% (v/v) ethanol with 10% (v/v) acetic acid, washed with water, and stained with CBB G-250. The best 3 reproducible runs of both larvae's gel images at 72 and 120 hours were used for further analysis. PDQuest V 8.0 (Bio-Rad) was used to analyze the data from 2-DE gels. The authenticity and outline of each spot was validated by visual inspection and edited if necessary.

In order to accurately compare spot quantities of the two samples, the volume of each protein spot was normalized and automatically calculated by the software as a single spot volume. After normalization and background subtraction, the matched sets were created for all the samples. A quantitative table with all the normalized optical spot volumes was generated in this software for subsequent statistic analysis. Analysis of variance (ANOVA, Version 6.12, SAS Institute), Duncan parametric test, was employed to test the statistic significance of the differentially expressed proteins (2 fold changes) in all gels. A probability of *p*<0.05 was considered to be statistically significant.

### Tryptic Digestion and Protein Identification by Mass Spectrometry

The differentially expressed proteins spots were manually excised from CBB stained gels. Gel fragments were digested with trypsin (Roche, Cat. No. 11418025001) for MS analysis according to our previously described method [20]. Matrix was prepared by dissolving α-cyano-4-hydroxycinnamic acid (CHCA, Bruker Daltonics) in 50% acetonitrile/0.1% trifluoroacetic acid. 10 µL of the matrix solution was added onto the dried digests and vortexed for 30 min. A total of 1.5 µL of the reconstituted in-gel digest sample was then spotted on Anchorchip target plate (600/384F, Bruker Daltonics), followed by addition of 1 µL matrix solution onto each spot. The dried sample on the target plate was washed twice with 1 µL of 0.1% TFA, left for 30 s before solvent removal, and dried for matrix assisted laser desorption ionization time of flight/mass spectrometry (MALDI TOF/MS) analysis on Autoflex MALDI TOF/TOF MS instrument (Bruker Daltonics). Peak-lists were generated by Mascot Distiller software (Version 3.2.1.0, Matrix Science). Parameters and thresholds used for peak picking were signal/noise threshold >6 and resolution >1500. The peptides for the calibration were done by a protonated mass signal from a standard peptide calibration mixture consisting of 8 peptides covering mass range from 700 to 3100 Da (Bruker, Billerica, MA Peptide Calibration Standard 206196) (Bruker daltonics). Then, the measured tryptic peptide masses were transferred through MS BioTool (Version 2.2, Bruker daltonics) as inputs to search against the non redundant NCBI (NCBInr) database using MASCOT 2.2 (Matrix Science). Search parameters were as follows: Taxonomy: all entries; trypsin cleavage; allow up to one missed cleavage; peptide mass tolerance 0.2 Da; fixed modification: carbamidomethyl (C); variable modification: oxidation (M).

When the peptides were matched to multiple members of a protein family, or a protein appeared under same names and accession number, the match was considered in terms of a higher Mascot score, the putative function and differential patterns on 2-DE gels. Protein identifications were accepted if they had greater than 95% probability and contained at least 2 identified peptides, maximum peptides coverage.

### Protein classification

The identified proteins were searched against the Uniprot database (http://www.uniprot.org/ ) and flybase (http://flybase.org/) for their functions. According to the combined searching results, these proteins were divided into different functional groups.

### Biological Network and Functional Enrichment Analysis

After obtaining the list of the differentially expressed proteins at 72 and 120 hours of the queen and worker larvae sample from the MALDI-TOF/MS analysis, proteins of interests were generated from this protein list. Proteins of interest were further analyzed by Pathway Studio software (Ariadne Genomics). Briefly, the protein list was blasted against the *Drosophila* database that was implemented with the functional relationships of protein molecules supported by the scientific literature. The filter, “all shortest paths between selected entities”, was applied and the data analysis information received was narrowed down to our protein list of interest, namely, proteins whose involvement and regulatory functions had been observed. Each link was built with evidence from at least 3 publications. Protein entities which belong to different functional groups were represented by different shapes according to the default settings of the software.

To enrich the identified proteins to specific functional terms, the protein list was analyzed by CluoGo software applying to the *Drosophila* database downloaded from the Gene Ontology database (release date, January 10, 2010) [21]. Ontology was selected as biological process. Enrichment analysis was done by right-side hypergeometric statistical testing and the probability value was corrected by Bonferroni method.

### Quantitative real-time PCR

Total RNA was extracted from queen and worker larvae at 72 and 120 hours using TRIzol regent (Takara bio). Reverse transcription was performed using a RNA PCR Kit (Takara bio), according to the manufacturer's instructions. Nine differentially expressed proteins were examined to detect the corresponding mRNA levels by quantitative real-time PCR, based on the sequences in honeybee cDNA library. Gene names, accession number, forward and reverse primer sequence and amplification size were listed and GAPDH (glyceraldehyde 3-phosphate dehydrogenase) was used as the reference gene (Table S1). Real-time PCR amplification was conducted on iQ5 Multicolor Real-Time PCR Detection System (Bio-Rad). Every sample was analyzed individually and processed in triplicate. Each reaction volume was performed in a total of 25 µl containing 1 µl of cDNA template and 5 pmol of primer, 12.5 µl SYBR Green Supermix (dNTPs, iTaq DNA polymerase, 6 mM MgCl_2_ , SYBR Green and 6.5 µl double distilled water, fluorescent and stabilizers, RNase-free water) (Bio-Rad). The real-time PCR was performed as 1 cycle of denaturation at 95°C/10 min, followed by 40 cycles of amplification (95°C/30s, 60°C/30s, 72°C/45s) where the fluorescence was automatically measured during PCR. The specificity of the amplified product was monitored using its melting curve. Gene expression data was normalized by GAPDH. After verifying amplification efficiency of the selected genes and GAPDH in approximately equal level, the difference in gene expression levels were calculated using the 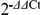 method (22).

## Results

### Identification of differentially expressed proteins

**Figure 1:**
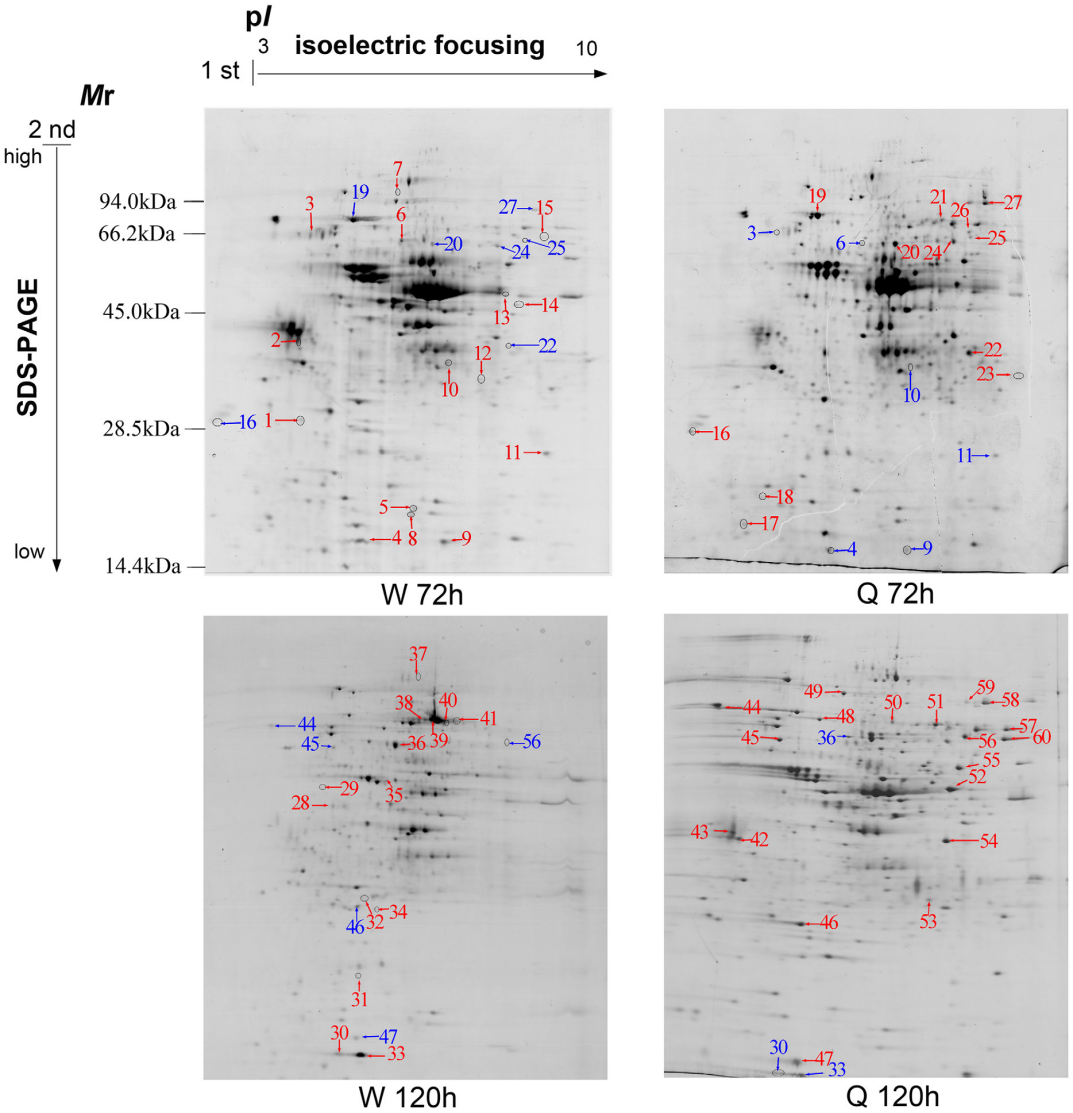
Proteome map of honeybee worker and queen larvae at developmental stages 72 hours (w 72 h and Q 72 h) and 120 hours (W 120 h and Q 120 h). 2-DE separation was performed on IPG gel strips (17 cm, 3- 10L) followed by SDS-PAGE on a vertical slab gel (12%). Protein spots of known identity are marked with color codes, red indicating upregulated proteins and blue indicating down regulated.

In order to investigate the involvement of critical primary proteins as well as to establish their influences in caste differentiation at an early post embryonic stage, proteomic profiles of queen and worker larvae were compared at 72 and 120 hours using 2-DE gels. In summary, a total of 288 and 259 protein spots were detected at 72 hours, and 274 and 236 protein spots at 120 hours in queen and worker larvae, respectively. Among these proteins, 59 spots at 72 hours and 51 spots at 120 hours were reproducibly detected with expression changing more than 2 fold between the two castes (*p*<0.05). Furthermore, at 72 hours 40 protein spots (worker 18 and queen 22) and at 120 hours 41 protein spots (worker 22 and queen 19) were successfully identified by MALDI TOF/MS for the queen and worker larvae, respectively. From all the identified proteins at 72 hours, 15 were upregulated in the worker larvae and 12 in the queen larvae, and likewise, at 120 hours 14 proteins were upregulated in the worker larvae and 19 in the queen larvae (Table S2, Figure 1).

### Qualitative and Quantitative Comparison of Differentially Expressed Proteins between Two Castes Larvae

**Figure 2:**
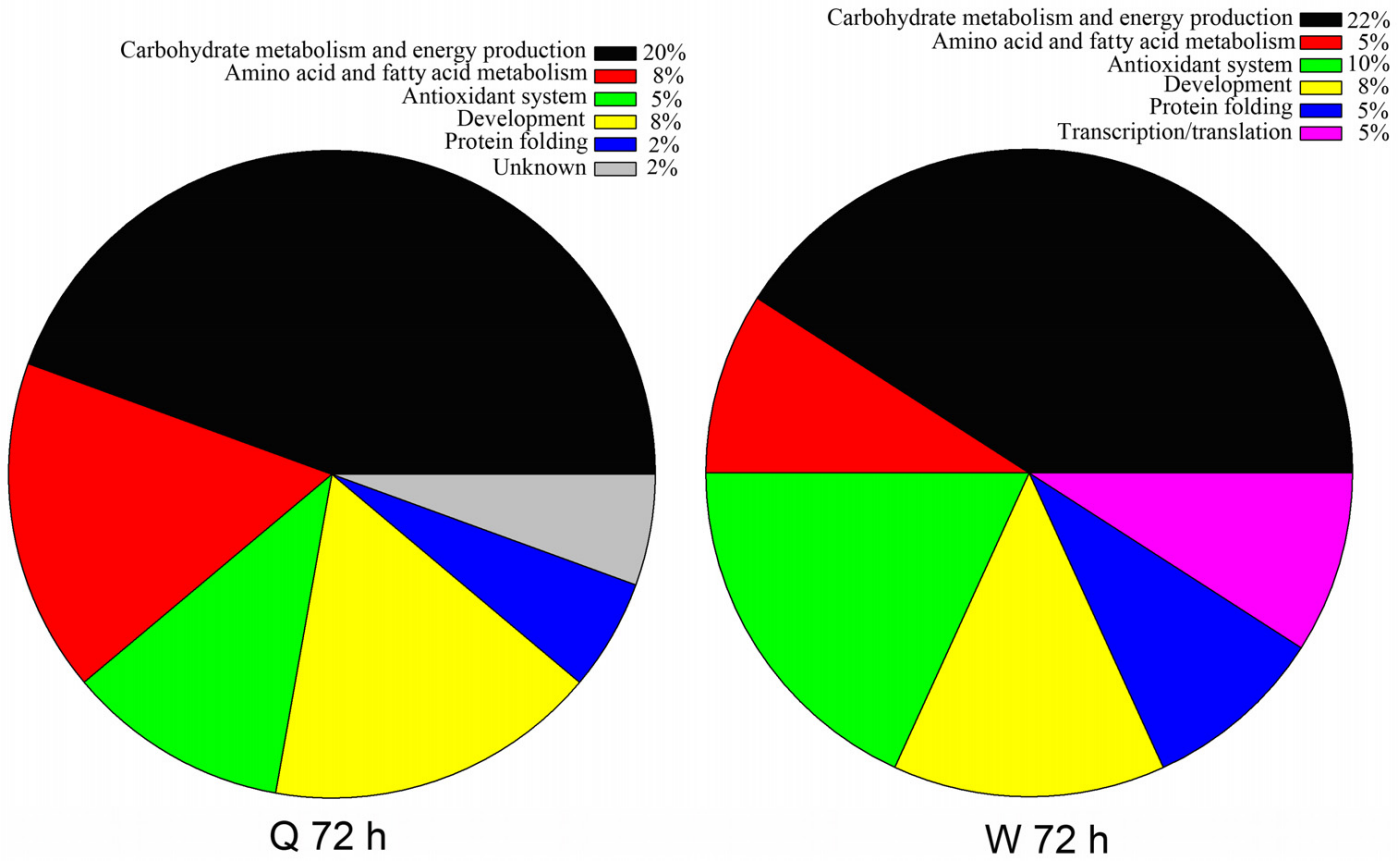
Functional distributions of the identified proteins from queen and worker larvae at 72 hours (Q 72 h and W 72 h) of developmental stage. The percentage of each functional group was obtained based on the calculated absolute number of proteins under each of the functional groups subtracted from the totally number of identified proteins at 72 hours (table S3).

**Figure 3:**
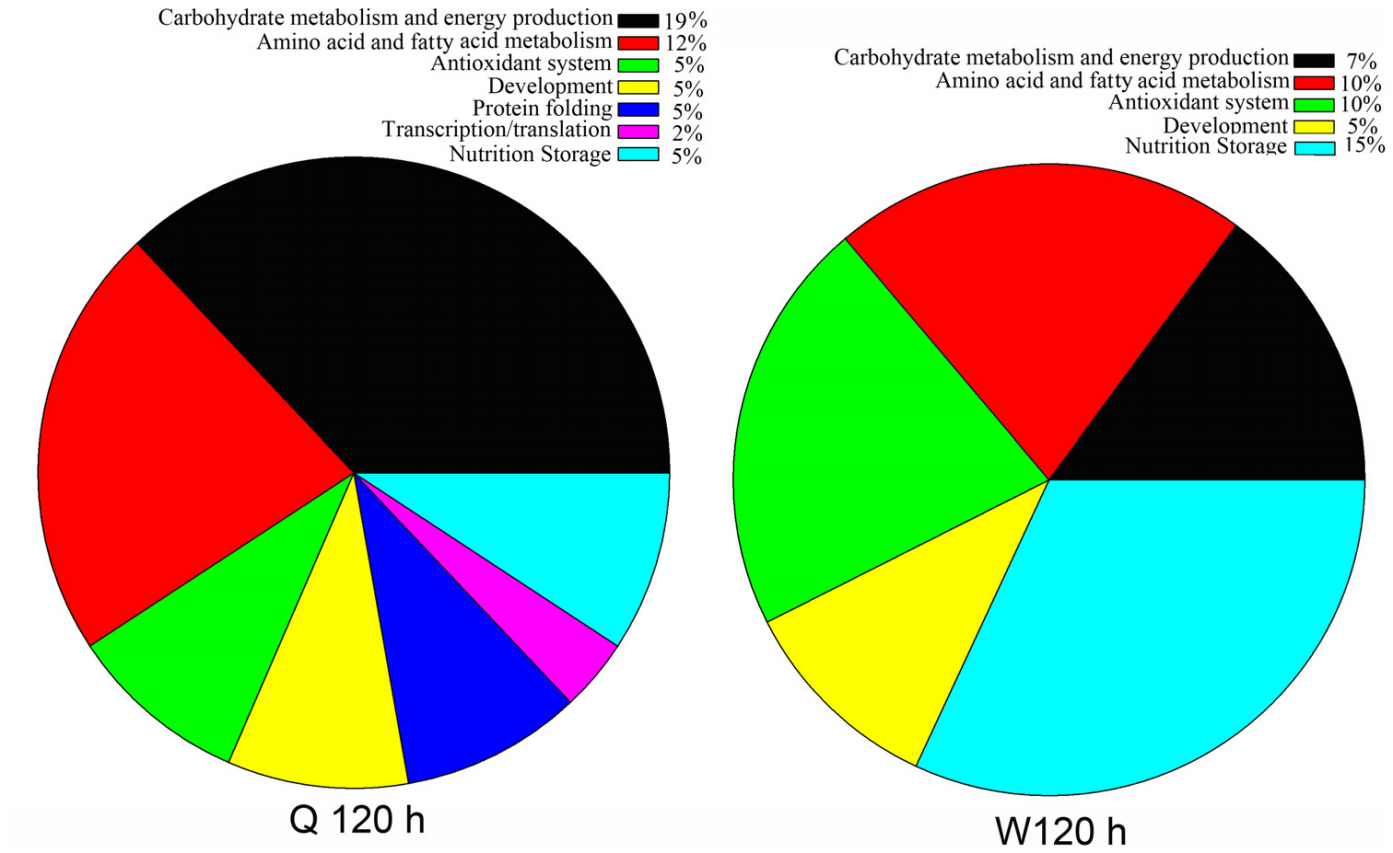
Functional distributions of the identified proteins from queen and worker larvae at 120 hours (Q 120 h and W 120 h) of developmental stage. The percentage of each functional group was obtained based on the calculated absolute number of proteins under each functional group subtracted from the totally number of identified proteins at 120 hours (table S4).

As shown in Figure 2, at 72 hours proteins involved in carbohydrate metabolism and energy production were the most represented category and qualitatively the proportion was almost the same in both larvae castes. However, proteins involved in amino acid and fatty acid metabolism, antioxidation, protein folding and transcription/translation showed qualitative differences. Proteins implicated in developmental related processes were present in equal proportions in both queen and worker larvae. Similarly at 120 hours, the proportional representations of different functional classes of the identified proteins showed variations over the two intended larvae castes. The representations of carbohydrate metabolism and energy production proteins in queen larvae remained constant as of the 72 hour developmental stages and drastically dropped in worker larvae (Figure 3).

From a qualitative perspective, the two larvae casts were compared based on the upregulated number of proteins at both developmental time-points. To do so, protein spots appeared on the gel with different numbers, but finally were confirmed as a single protein and were considered only one time. This is most likely due to the occurrence of some post-translational modifications (PTMs) for some proteins, such as phosphorylation and possibly alternative splicing or proteolysis that results in the shifting of *M*r and p*I* of these proteins. Hence, 18 and 22 individual protein spots were recognized as upregulated at 72 and 120 hours, respectively in both larvae. Out of the upregulated proteins at 72 hours, both queen and worker larvae upregulated the same 11 individual proteins. From the 11 proteins in queen larvae, 5 were involved in carbohydrate metabolism and energy production (enolase spots 20, aldehyde reductase, spot 22, phosphoglycerate mutase, spot 23, Bellwether, spot 25 and transketolase, spot 27), 2 for metabolizing fatty acids and amino acids (long-chain-fatty-acid CoA ligase, spot 21 and proteasome subunit alpha type 5, spot 16), 1 in development (imaginal disc growth factor 4, spots 24, 26), 1 for antioxidation (thioredoxin peroxidase 1, spot, 18), 1 for protein folding (heat shock protein 8, spot 19) and 1 protein of unknown function (GE14749, spot 17) (Table S2). Meanwhile, out of the 11 worker larvae upregulated proteins, 4 were involved in carbohydrate metabolism and energy production (aldehyde dehydrogenase, spots 6 and 13 and ATP synthase beta subunit, spot 3, phosphoglycerate mutase, spot 12, Bellwether, spot 11), 1 in fatty acid metabolism (fatty acid binding protein, spot 4), 2 antioxidant protein (peroxiredoxin 2540, spot 9, thioredoxin peroxidase 1, spots 5, 8, 14), 2 for development (14-3-3 protein epsilon, spot 2 and lethal (2) 37, spot 10) 1 in protein folding (heat shock protein 8, spot 7) and 1 in transcription/translation (translational controlled tumor protein spot 1, 15) (Table S3).

Similarly, from the 22 individual proteins upregulated at 120 hours, 16 and 9 were in the queen and worker larvae, respectively. Out of the 16 upregulated proteins in queen larvae, 6 were involved in carbohydrate metabolism and energy production (enolase, spots 42, phosphoglycerate mutase, spot 53, phosphoglycerate kinase, spot 60, ATP synthase beta subunit, spot 45, transketolase, spots 58, 59 and aldehyde dehydrogenase, spot 52, 54), 2 for metabolizing fatty acids and amino acids (long-chain-fatty-acid CoA ligase, spots 51 and ornithine aminotransferase precursor, spot 55), 2 for antioxidant protein (Glutathione S transferase S1, spot 46, thioredoxin peroxidase 1, spot 47), 2 for development (Leonardo, spot 43 and imaginal disc growth factor 4, spot 56), 2 for protein folding (ERp60, spot 48) and heat shock protein 8, spot 49), 1 for transcription/translation (translational controlled tumor protein, spot 57) and 1 nutrition storage protein (larval serum protein 1, spots 44, 50) (Table S4).

Meanwhile, out of the 9 upregulated proteins in worker larvae at 120 hours, 2 were for carbohydrate metabolism and energy production (ATP synthase beta subunit, spot 34 and transketolase (spot 36), 2 were involved in amino acid and fatty acid metabolism (arginine kinase, spot 35 and fatty acid binding protein, spots 30, 33), 2 antioxidant proteins (thioredoxin peroxidase 1, spot 31, short-chain dehydrogenase/reductase, spot 32), 2 for development (cathepsin D, spot 28 and actin-87E isoform 2, spot 29) and 1 nutrition storage protein, (larval serum protein 2, spots 37–41). (Table S4).

**Figure 4:**
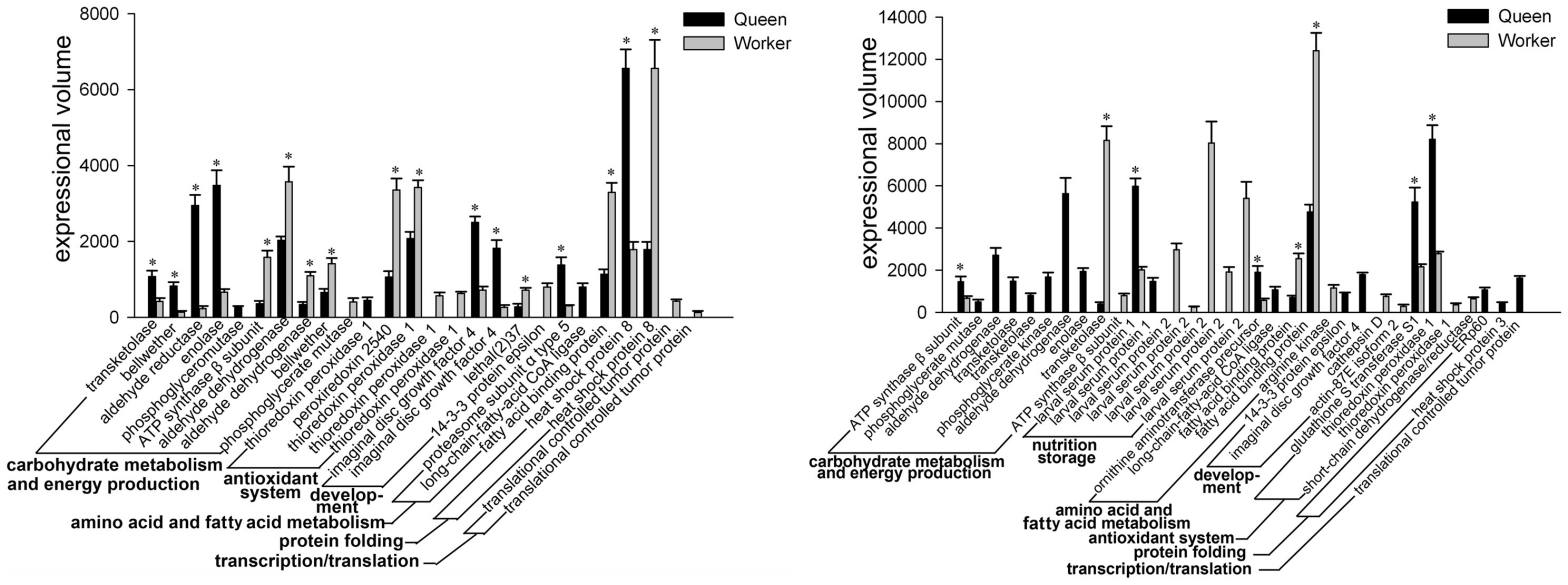
Comparison of protein expression levels in castes over 72 and 120 hours developmental stages, where the black bars denote the protein expression levels in queen larvae and grey bars denote the protein expression levels in worker larvae. An asterisk at the top of the bars indicates the existence of statistically significant differences in the expressional level.

In addition to qualitative differential protein expression, the two larvae were also compared based on quantitative differential protein expression levels. As the two larvae upregulated some important proteins in common at both developmental stages, this method is particularly useful to support the results of the qualitative studies. Accordingly, the expression level for most of the functional classes at both developmental stages showed differences between the two larvae. There was over-expression in queen larvae at 72 hours in transketolase, aldehyde reductase, enolase, phosphoglycerate mutase, imaginal disc growth factor 4, proteasome subunit alpha type 5 and long-chain-fatty-acid CoA ligase as compared with the worker larvae. Likewise, worker destined larvae over-expressed ATP synthase ß subunit, aldehyde dehydrogenase, phosphoglycerate mutase, peroxiredoxin 2540, thioredoxin peroxidase 1, lethal (2)37, 14-3-3 protein epsilon and fatty acid binding protein (Figure 4). At 120 hours, queen larvae over-expressed phosphoglycerate mutase, aldehyde dehydrogenase, phosphoglycerate kinase, enolase, larval serum protein 1, ornithine aminotransferase precursor, long-chain-fatty-acid CoA ligase, 14-3-3 protein epsilon, imaginal disc growth factor 4, glutathione S transferase S1, thioredoxin peroxidase 1, ERp60, heat shock protein 3 and translation controlled tumor protein as compared with the worker larvae. Worker larvae over-expressed larval serum protein 2, fatty acid binding protein, arginine kinase, cathepsin D, actin-87E isoform 2 and short chain dehydrogenase/reductase l as compared to the queen larvae (Figure 4).

### Functional Enrichment and Biological Network Analysis

**Figure 5:**
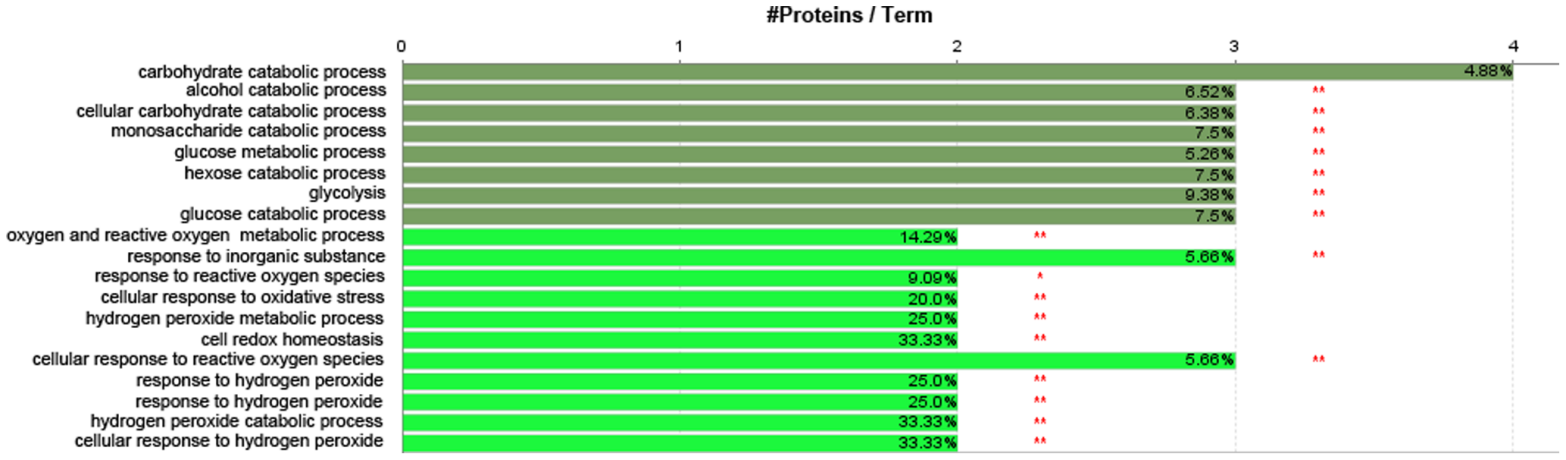
ClueGO software analysis output indicating the functional term enrichments of the identified proteins to carbohydrate metabolism and energy production, and antioxidant proteins, where the deep green color denotes carbohydrate metabolism and energy production and the light green color denotes antioxidants.

Gene Ontology (GO) annotation, which provides 3 detailed, structured terms including molecular functions, biological processes, and cellular components, are now widely used in the analysis of large proteomic and genomic datasets, where significantly overrepresented GO terms are examined to determine hypotheses for the biological events behind the data and to provide a broad overview of the principal characteristics of a proteome. In order to enrich the identified proteins to specific functional terms, protein lists were analyzed by CluoGo software. Accordingly, our protein list was significantly enriched to two major functional groups i.e., carbohydrate metabolism and energy production, and antioxidant proteins (Figure 5). The leading term in carbohydrate metabolism and energy production was carbohydrate catabolic process in which transketolase, enolase, phosphoglycerate kinase, phosphoglycerate mutase were significantly enriched. And the leading term in antioxidant was peroxiredoxin 2540 and thioredoxin peroxidase 1.

**Figure 6:**
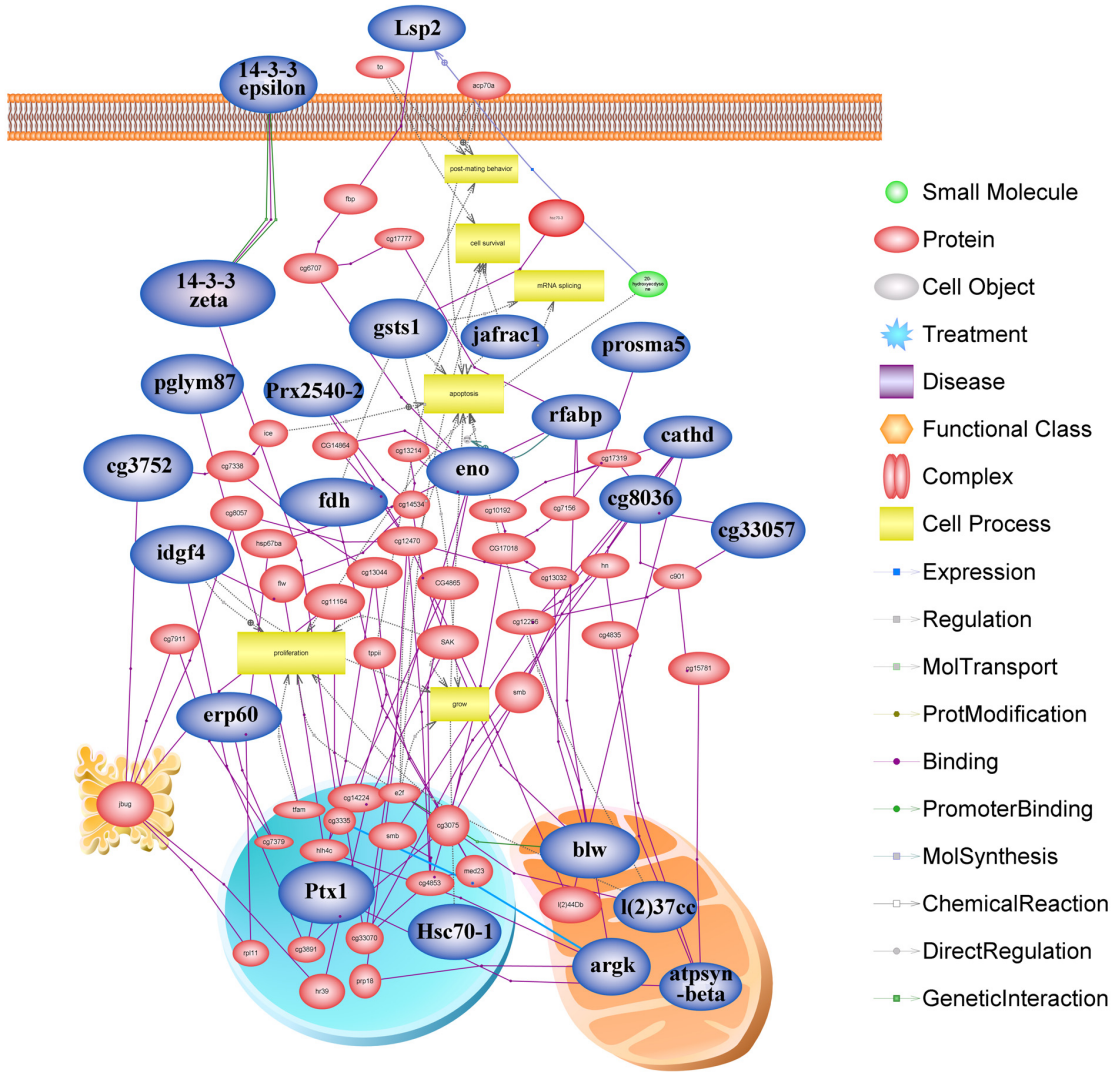
Network analysis of all the pathways and interactions connected to all of the identified proteins. Those highlighted in blue represent the key node proteins identified in this study and validated by the network software program. Protein entities which belong to distinct functional groups were represented as different shapes according to the default settings of the software as described in the legend.

In a living cell proteins perform their functions not as a single entity but rather work together in the context of networks. We analyzed all of the pathways and interactions connected to each of the identified proteins hoping to find the possible key node proteins during the larval development using pathway studio software. As a result, 23 proteins classified into 6 functional groups were linked in the network based on diverse linkage relationships like protein-protein interactions, modifications and regulation of expression (Figure 6). Based on the protein functional group, most proteins presented in the network (30%) were related to carbohydrate metabolism and energy production, namely, aldehyde dehydrogenase (cg3752), aldehyde reductase (fdh), ATP synthase beta subunit (atpsynbata), Bellwether (blw), enolase (eno), phosphoglycerate mutase (pglym87) and transketolase (cg8036). Proteins involved in development processes were the second most represented (20%) and comprise Leonardo (14-3-3 zeta), 14-3-3 protein epsilon (14-3-3 epsilon), actin-87E isoform 2 (Ptx1), cathepsin D (cathd), imaginal disc growth factor 4 (idgf4) and lethal (2)37 (l(2)37cc). Proteins involved in metabolizing amino acid accounted for 9% that included proteasome subunit alpha type 5 (prosma5), arginine kinase (argk), and 4% fat acid metabolism, (fatty acid binding protein, rfabp). As the 2^nd^ most represented protein in the functional enrichments (13%), antioxidant proteins comprised peroxiredoxin 2540 (prx2540-2), glutathione S transferase S1 (gsts1) and thioredoxin peroxidase 1 (jafrac1). Protein folding was also the 3^rd^ represented (9%) functional term in the network linked through heat shock protein 8 (Hsc70-1) and ERp60 (erp60). Whereas, nutritional storage and transcription/translation proteins were networked by larval serum protein 2 (Lsp2) and translational controlled tumor protein (cg33057), respectively (Figure 6).

**Figure 7:**
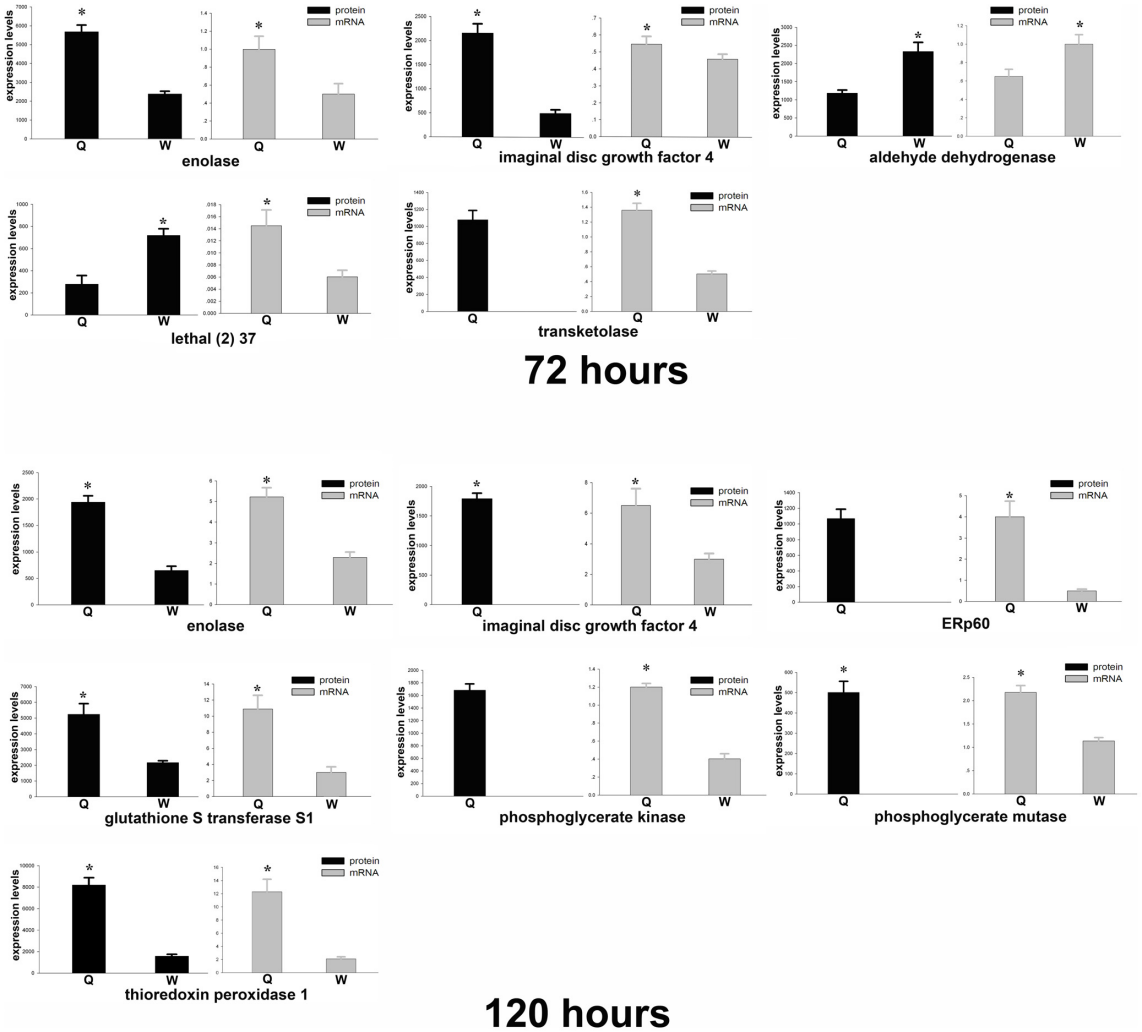
Validation of protein expression at mRNA level by quantitative real time RT-PCR analysis. Samples were normalized with the Gapdh gene as the control house-keeping gene. Error bars represent the standard deviation.

Furthermore, protein expression differences between the two castes were validated at mRNA levels for 10 selected key node proteins in the network. Accordingly, proteins as transketolase, aldehyde dehydrogenase, and lethal (2) 37 at 72 hours, phosphoglycerate mutase, glutathione S transferanse S1, ERp60, phosphoglycerate kinase and thioredoxin peroxidase 1 at 120 hours, and enolase and imaginal disc growth factor 4 and were chosen at both time points. Generally, the changes in mRNA abundance of the genes at 72 hours of the two castes were similar to that of the 2-DE data, except for the transcripts of thioredoxin peroxidase 1 and lethal (2) 37 (Figure 7). Among these genes, mRNA abundance of enolase, transketolase and imaginal disc growth factor 4 genes were increased in queen larvae, and aldehyde dehydrogenase were increased in worker larvae. Likewise, at 120 hours, mRNA quantification further validated the protein differential expression results in the two larvae (Figure 7). In summary, 7 genes; namely, enolase, phosphoglycerate mutase, phosphoglycerate kinase, imaginal disc growth factor 4, thioredoxin peroxidase 1, ERp60 and glutathione S transferase S1, were constantly upregulated at both protein and mRNA levels in queen larvae. The discrepancy of thioredoxin peroxidase 1 and lethal (2) 37 in their mRNA and protein level at 72 hours, is most likely due to the lack of a direct relationship between mRNA and protein expression, and/or other regulatory mechanisms, such as lack of synchronization. These data provide transcriptional information complementary to the differentially expressed proteins detected by proteomics analysis.

## Discussion

To uncover the underlying mechanisms of honeybee caste differentiation, this study compared the global proteome of worker and queen larvae of the honeybee at 72 and 120 hours developmental stages. The results provide preliminary information at the protein level for future advanced molecular and biochemical investigations of caste pathway decisions on the honeybee and other related eusocial insects. The results of the proteome comparison between both queen and worker caste destined larvae at two developmental stages showed that proteins involved in carbohydrate metabolism and energy production were the most upregulated proteins. This suggests that both larvae at the fast growth stage demand high energy source proteins. The results of the enrichment and biological networks analysis confirmed that proteins for antioxidation were the second most upregulated proteins and played significant roles in the formation of caste polymorphism.

There was a significant difference between the two caste destined larvae in quantitative protein expressions at 72 hours, suggesting that the difference in biological requirements that lead the two larvae into caste divergence has already began. This is in contrast to previous studies [8], [23] which support the notion that day 3 larva is totipotent and are able to differentiate into either worker or queen. The divergences of the two caste destined larvae became more profound at 120 hours through quantitatively and qualitatively upregulated proteins. Qualitatively, the worker larvae upregulated proteins were reduced from 11 to 9, while the number increased significantly in queen destined larvae from 11 to 16, confirming the divergence [3], [24] dictated and directed by specific proteins types and amounts. In particular, the variations between the two larvae in terms of proteins for carbohydrate metabolism and energy production increased their metabolic activities at both developmental stages significantly. More specifically, the over-expression of transketolase, aldehyde reductanse and enolase at 72 hours in queen destined larvae and ATP synthase beta subunit and phosphoglycerate mutase in worker destined larvae suggests that the two larvae differed in their glucose level and saccharidase activity at this or an earlier stage. Moreover, out of the 6 upregulated proteins related to carbohydrate metabolism and energy production in the queen destined larvae, 2 proteins were upregulated in worker destined larvae at late stages of 120 hours. In support of this, important carbohydrate metabolism and energy production metabolic proteins like aldehyde dehydrogenase, transketolase, ATP synthase ßsubunit, phosphoglycerate mutase and enolase showed considerable age-linked increase in queen destined larvae. Together with functional enrichment and biological network analysis, our data suggests that carbohydrate metabolism and energy production proteins play special and central roles in caste differentiation and determination [23], [25], [26]. Moreover, especially for the queen larvae, phosphoglycerate kinase and aldehyde dehydrogenase became apparent only at 120 hours and enolase, phosphoglycerate mutase and transketolase were present at both developmental stages. The particular over-expression of these specific proteins in the queen intended larvae indicate their explicit function towards enhancing the metabolic energy requirement of queen larvae to assist in attaining queen distinctive physiological and behavioral characters [23]. The metabolism of aldehyde dehydrogenase has been already reported selectively in the queen mandible [27] and its discriminatory occurrence in queen destined larvae in this study is most likely to equip the mandible of the future queen with special functions like production of pheromonal signals that involve the regulation of the reproductive hierarchies [28]. Moreover, aldehyde dehydrogenase involved in glycolysis pathways together with transketolase involved in pentose phosphate pathway were observed as changing protein copy number. Hence, as changes in gene copy number are a vital source of variation and driving force in phenotypic evolution [29], [30], likewise, we consider their over-expression as motivators for metabolic activities in queen larvae towards attaining distinctive phenotypic features [1]–[3], [17].

Amino acid and fatty acid metabolizing proteins are vital for larval rapid growth and are used in the process of metamorphosis [3] and tissue construction [31]. Their upregulation in worker larvae at late developmental stages and in queen larvae at early developmental stages agree with previous studies [3], [19], [26], [32] Furthermore, the over-expression of arginine kinase and fatty-acid binding protein which function as an energy transporter from mitochondria to high energy demanding areas for the development of honeybee brain, thorax, compound eye and antennae, during the late developmental stage in worker larvae [33] is in agreement with previous reports [3], [31]. The upregulation of long-chain-fatty-acid CoA ligase only in queen destined larvae at both developmental stages suggests a high demand for lipid metabolizing enzymes to cope with the high metabolic rate and development of queen destined larvae via food degredation to the release of stored energy and synthesis of primary stored energy.

Reactive oxygen species (ROS) cause oxidative damage especially in a fast growing organism which demand high oxygen levels [34]. Peroxiredoxin 2540 and thioredoxin peroxidase 1 function as peroxide metabolizers [35]. It has been reported that expression of antioxidant genes generally increases in the younger queens [36] and high demand for oxygen during embryonic development leads to increased level of ROS and thus increasing oxidative damage [37], [38]. Also, it has been documented that antioxidants play important defensive roles in the development of the honeybee embryo [39] and hypophrigeal gland [40] as well as in facilitating long-term storage of sperm in the spermathecal fluid [41]. The over-expression of peroxiredoxin 2540 and thioredoxin peroxidase 1 at 72 hours in worker larvae and thioredoxin peroxidase 1 and glutathione S transferase S1 at 120 hours in queen larvae suggest a key role of the antioxidant system in both developing larvae by changing oxidation products into harmless chemicals [42].

The upregulation of imaginal disc growth factor 4 in queen larvae at both developmental stages supports formation of imaginal disc cells, which in turn accelerates organogenesis before the transformation of larvae into pupae [7]. Despite its recognition in developing larvae [7] as well as in the hemolymph of freshly emerged adult worker bee [26], imaginal disc growth factor 4 was totally missing in worker destined larvae in this study, which might be due to too low abundance to be detected by 2-DE and/or differences in protein extraction methods. The expressions of Leonardo protein that is known to be involved with the formation of olfactory learning and memory structures at late developmental stages in only queen larvae suggests the beginning of the formation of these important structures to equip the future queen with the sensory cells necessary for scent recognition and learning abilities [9], [23]. However, the expression of actin-87E isoform 2 only in worker larvae at 120 hours is most likely to assist the embryonic development dorsal closure as it was known to function in the *Drosophila* [43].

Proteins with binding function like the heat-shock protein family are known to play vital roles in apoptosis reduction [44]. However, as our larvae samples were grown in a normal colony, the presently identified ERp60 and heat shock protein 8 are most likely to act as molecular chaperones in a living cell to make sure that the cellular proteins could function correctly [26], [40]. Interestingly, both larvae did not upregulate nutritional storage proteins at 72 hours, however were over-expressed at 120 hours in both. This is in line with the case for *Drosophila* which exhibited increased expression of larval serum protein 2 during the 3rd larval instar through midway to the pupal stage for the subsequent proteolytically degraded amino acid supply, which are necessary activities for the completion of pupae and adult development [45], [46].

The honeybee genome has been sequenced and RNA interference has already proven to be a successful tool for *in vivo* studies of gene function and phenotypes [47], [48]. Based on the good validation of the results between proteins and genes in the network, we also see interesting potential in reverse genetics that indicate the manipulation of genes could determine the fate of the developing larvae.

### Conclusions

The data reported in this study reveal the differential protein expression profile of queen and worker intended larvae at 72 and 120 hours of developmental stages. It is the first report that the two caste destined larvae show apparent differences in protein expressional levels as early as 72 hours. Molecularly, the distinctive biological requirements in queen intended larvae was driven by aldehyde reductase and enolase, imaginal disc growth factor 4, long-chain-fatty-acid CoA ligase and proteasome subunit alpha type 5. Whereas, in the worker intended larvae the distinctive biological requirements was driven by ATP synthase beta subunit, aldehyde dehydrogenase, thioredoxin peroxidase 1, peroxiredoxin 2540, lethal (2) 37, 14-3-3 protein epsilon, fatty acid binding protein and translational controlled tumor protein. The divergence between the two caste intended larvae was more profound at 120 hours as evidenced by increased over-expression of important and caste unique proteins. The influences of the over-expression of these particular proteins concerning the caste direction were confirmed at the mRNA level. More specifically, the over-expression of aldehyde reductase at 72 hours, and phosphoglycerate kinase and aldehyde dehydrogenase at 120 hours, and enolase, transketolase and imaginal disc growth factor 4 at both time points in queen larvae identify them as caste specific and possible proteins for the queen intended larvae to follow a different developmental trajectory.

To summarize, based on the proteomic assessment, we report that day 3 and day 5 queen and worker larvae have different protein profiles and different biological requirements that lead them to follow distinct caste trajectories. This study indicates that 3-day old larva is already on the pathway for the intended direction. Therefore, further studies will target earlier larvae ages (less than day 3) for detail investigations on molecular mechanisms underlying caste divergence at early post embryonic developmental stage.

## Supporting Information

Table S1 Primer sequences used for validating real-time PCR of genes expressed during the larval development of honeybee drone (Apis mellifera L.).
(0.06 MB DOC)

Table S2 Expressed protein honeybee worker and queen larvae proteins by functional category, spot codes and status at 72 and 120hrs.
(0.07 MB DOC)

Table S3 Differentially expressed proteins of honeybee worker and queen larvae at 72 hours.
(0.09 MB DOC)

Table S4 Differentially expressed proteins of honeybee worker and queen larvae at 120 hours.
(0.10 MB DOC)
