# Supplementary material for: Differential Protein Expression in Honeybee (Apis mellifera L.) Larvae: Underlying Caste Differentiation
Source: PLoS One. 2010 Oct 20;5(10):e13455. doi: 10.1371/journal.pone.0013455 (PMC2958119; doi:10.1371/journal.pone.0013455)
Supplement: Table S1 — Primer sequences used for validating real-time PCR of genes expressed during the larval development of honeybee drone (Apis mellifera L.). (0.06 MB DOC) [file pone.0013455.s001.doc]

**Table S1. Primer sequences used for validating real-time PCR of genes expressed during the larval development** of honeybee drone (Apis mellifera L.)

| Accession  Number | Spot No. | Protein Name | Primer Sequences | Sense 5’-3’ | Product  Size (bp) | Annealing  Temperature(˚C) |
| --- | --- | --- | --- | --- | --- | --- |
|  |  |  |  | Antisense 5’-3’ |  |  |
| gi|66530423 | 6, 13 | aldehyde dehydrogenase | GTATAGCCACATTGCGATATTATG | | 160 | 59.0 |
|  |  |  | TTCCAAGCCATCATTAAGATAGG | |  |  |
| gi|66514614 | 24, 26 | imaginal disc growth factor 4 | CCAACCTTACAACGAGAACAC | | 186 | 59.0 |
|  |  |  | GCGGCTCTTATTATAGGATACTTG | |  |  |
| gi|48097857 | 10 | lethal (2) 37Cc | ACCAAGTTGTAGGAGAAGGAAC | | 151 | 59.0 |
|  |  |  | CTGAAGAGAATACGAAGTGTGATG | |  |  |
| gi|66546657 | 48 | ERp60 | AACTTGCGAATGAGGATGTTGAG | | 150 | 59.0 |
|  |  |  | GCTCTCTGCCACCTTCATATTTG | |  |  |
| gi|110751363 | 27, 36,  58, 59 | transketolase | TGCTACAAGACTGGCTTATGG | | 182 | 59.0 |
|  |  |  | CACCGATTGCTACTCCTACTAC | |  |  |
| gi|110761968 | 20, 42 | enolase | ACTTGGATGTTACTTCACAATCTG | | 129 | 59.0 |
|  |  |  | CTGCTCCTGCTTTACAAACTG | |  |  |
| gi|66550890 | 12, 23,53 | phosphoglycerate mutase | GCTGAAACTGCTGCTAAATATG | | 78 | 59.0 |
|  |  |  | CATAGGTGGAGGAGGTGTATC | |  |  |
| gi|66548188 | 5, 8,14, 18,  31, 47 | thioredoxin peroxidase 1 | CAGCATCTACTGATTCTCACTTC | | 166 | 59.0 |
|  |  |  | AGACCACGAAATGGAACTCC | |  |  |
| [gi|110763826](http://www.matrixscience.com/cgi/protein_view.pl?file=../data/20080925/FtTcrnuST.dat&hit=1) | 60 | phosphoglycerate kinase | CTATAATTGGTGGTGGTGATACAG | | 145 | 59.0 |
|  |  |  | AACGAAGATAAAGCAGCAACTC | |  |  |
| [gi|66534655](http://www.matrixscience.com/cgi/protein_view.pl?file=../data/20070925/FtncmfTaS.dat&hit=1) | 46 | glutathione S transferase S1 | AAGTGAAGAATAATGGTGGCTAC | | 198 | 59.0 |
|  |  |  | CAGAATGTGGACGCTTATCAAG | |  |  |

Spot number corresponds to the number of protein spots in Figure 1 and Table S3-S4.
