# Supplementary material for: Differential Protein Expression in Honeybee (Apis mellifera L.) Larvae: Underlying Caste Differentiation
Source: PLoS One. 2010 Oct 20;5(10):e13455. doi: 10.1371/journal.pone.0013455 (PMC2958119; doi:10.1371/journal.pone.0013455)
Supplement: Table S2 — Expressed protein honeybee worker and queen larvae proteins by functional category, spot codes and status at 72 and 120hrs. (0.07 MB DOC) [file pone.0013455.s002.doc]

**Table S2. Expressed protein honeybee worker and queen larvae proteins by functional category, spot codes and status at 72 and 120hrs**

| **Protein function**  **category** | **Caste**  **type** | **Developmental stages** | | | | | | | | **Grand total (spots)** |
| --- | --- | --- | --- | --- | --- | --- | --- | --- | --- | --- |
|  |  | **72 hours** | | | | **120 hours** | | | |  |
|  |  | **Spot code number** | **Total spots** | **Up regulation** | **Down regulation** | **Spot code number** | **Total spots** | **Up regulation** | **Down regulation** |  |
| Carbohydrate metabolism and energy production | Worker | 3,6,11,12,13,20,22,25,27 | 9 | 5 | 4 | 34,36,46 | 3 | 2 | 1 | 12 |
|  | Queen | 3,6,11,20,22,23,25,27 | 8 | 5 | 3 | 36,42,45,52,53,54,59,60 | 8 | 7 | 1 | 16 |
| Amino acid and fatty acid metabolism | Worker | 4, 16 | 2 | 1 | 1 | 30,33,35,55 | 4 | 3 | 1 | 6 |
|  | Queen | 4,16,21 | 3 | 2 | 1 | 30,33,51,55,58 | 5 | 3 | 2 | 8 |
| Antioxidant system | Worker | 5,8,9,14 | 4 | 4 |  | 31,32,46,47 | 4 | 2 | 2 | 8 |
|  | Queen | 9,18 | 2 | 1 | 1 | 46,47 | 2 | 2 |  | 4 |
| Development | Worker | 2,10,24 | 3 | 2 | 1 | 28,29 | 2 | 2 |  | 5 |
|  | Queen | 10,24,26 | 3 | 2 | 1 | 43,56 | 2 | 2 |  | 5 |
| Protein folding | Worker | 7,19 | 2 | 1 | 1 |  |  |  |  | 2 |
|  | Queen | 19 | 1 | 1 |  | 48,49 | 2 | 2 |  | 3 |
| Transcription/  translation | Worker | 1,15 | 2 | 2 |  |  |  |  |  | 2 |
|  | Queen |  |  |  |  | 57 | 1 | 1 |  | 1 |
| Nutrition Storage | Worker |  |  |  |  | 37,38,39,40,41,44 | 6 | 5 | 1 | 6 |
|  | Queen |  |  |  |  | 44,50 | 2 | 2 |  | 2 |
| Unknown | Worker |  |  |  |  |  |  |  |  |  |
|  | Queen | 17 | 1 | 1 |  |  |  |  |  | 1 |
| Worker | Total |  | 22 | 15 | 7 |  | 19 | 14 | 5 | 41 |
| Queen | Total |  | 18 | 12 | 6 |  | 22 | 19 | 3 | 40 |
| **Grand total** | |  | **40** | **27** | **13** |  | **41** | **33** | **8** |  |

Spot code numbers are the identification number given to each protein spot and corresponds to the number on the 2-DE gel at both developmental time points; Total spots indicates the total number of spots identified as expressed on the 2-DE gel; Upregulated indicates the amount of protein spots upregulated from the total expression; Down regulated indicates the amount of protein spots down regulated from the total expression.
