# Supplementary material for: Differential Protein Expression in Honeybee (Apis mellifera L.) Larvae: Underlying Caste Differentiation
Source: PLoS One. 2010 Oct 20;5(10):e13455. doi: 10.1371/journal.pone.0013455 (PMC2958119; doi:10.1371/journal.pone.0013455)
Supplement: Table S3 — Differentially expressed proteins of honeybee worker and queen larvae at 72 hours. (0.09 MB DOC) [file pone.0013455.s003.doc]

**Table S3. Differentially expressed proteins of honeybee worker and queen larvae at 72 hours**

| Spot  Number | Experimental  p*I* /*M*r(kDa) | Theoretical  p*I* /*M*r(kDa) | Sequence Coverage | Matched/Searched | Score | Protein Name | Accession Number | Up/down Regulated | Molecular function |
| --- | --- | --- | --- | --- | --- | --- | --- | --- | --- |
| **Carbohydrate metabolism and energy production** | | | | | | | | | |
| 12^W^  23^Q^ | 7.8/32.5  9.0/36.3 | 8.40/28.94  9.36/35.34 | 36.0%  35.0% | 10(59)  10(21) | 98  103 | Phosphoglycerate mutase (EC 5.4.2.1) | gi|66550890 | A  B | [2,3-bisphosphoglycerate-dependent phosphoglycerate mutase activity](http://www.ebi.ac.uk/ego/DisplayGoTerm?id=GO:0046538) |
| 13^W^  6^C^ | 8.0/74.5  6.3/62.2 | 6.69/55.65  6.69/55.65 | 22.0%  50.0% | 9(20)  25(59) | 103  168 | Aldehyde dehydrogenase (EC 1.2.1.5) | [gi|66530423](http://www.ncbi.nlm.nih.gov/blast/Blast.cgi?ALIGNMENTS=50&ALIGNMENT_VIEW=Pairwise&AUTO_FORMAT=Semiauto&CDD_SEARCH=on&CLIENT=web&COMPOSITION_BASED_STATISTICS=on&DATABASE=nr&DESCRIPTIONS=100&ENTREZ_QUERY=(none)&EXPECT=10&FILTER=L&FORMAT_BLOCK_ON_RESPAGE=None&FORMAT_OBJECT=Alignment&FORMAT_TYPE=HTML&GAPCOSTS=11+1&I_THRESH=0.001&LAYOUT=TwoWindows&MATRIX_NAME=BLOSUM62&NCBI_GI=on&PAGE=Proteins&PROGRAM=blastp&QUERY=MLRLLKKVRLSRYFSTATRPEPERNPAILYTGIFIDNEWHRSKSGKTFPTINPTTGETIAEIQEGDDADIDLAVNAANKAFKLGSPWRTMDASQRGVLLNNLASLMERHRAYLAALETLDNGKPYSDAYEFDVPSSIATLRYYAGWADKNHGQVIPIDGKYLAYTRHEPVGVCGQIIPWNFPILMMAWKLGPALATGNVIVLKPAEQTSLTALYIAQLCKDAGFPPGVINVVPGFGKTGAALVAHNLVDKIAFTGSTEVGKLIKQGAAMSNLKRTTLELGGKSPNIILSDVNLDQAVEAAHFGLFYNMGQCCCAGSRTFVEDSIYDEFVERSAARAKSRVVGNPFDSNVEQGPQIDEEQVNKIMSMIESGKNEGAELVSGGTRIGDKGYFVAPTVFANVKDYMTIAKEEIFGPVQQILKFSSLNEVITRANNTDYGLAAAVFTKDIDKANYIIQGLRAGTVWVNAYNVLTPQVPFGGFKMSGHGRELGQYGLEAYTEVKSVIVKVNQKNS&SERVICE=plain&SET_DEFAULTS.x=9&SET_DEFAULTS.y=5&SHOW_OVERVIEW=on&WORD_SIZE=3&END_OF_HTTPGET=Yes) | A  A | [aldehyde dehydrogenase (NAD) activity](http://www.ebi.ac.uk/ego/DisplayGoTerm?id=GO:0004029) |
| 3^C^ | 7.85/64.7 | 5.25/55.10 | 48.0% | 20(43) | 180 | ATP synthase beta subunit (EC [3.6.3.14](http://www.expasy.org/enzyme/3.6.3.14)) | gi|110762902 | A | [hydrogen ion transporting ATP synthase activity, rotational mechanism](http://www.ebi.ac.uk/ego/DisplayGoTerm?id=GO:0046933) |
| 11^C^  25^C^ | 8.4/23.6  8.2/68.4 | 9.00/59.51  9.00/59.76 | 33.6%  23.0% | 16(75)  16(70） | 120  313 | Bellwether | [gi|48100966](http://www.matrixscience.com/cgi/protein_view.pl?file=../data/20081127/FtTpmxcST.dat&hit=1) | A  B | [hydrogen ion transporting ATP synthase activity, rotational mechanism](http://www.ebi.ac.uk/ego/DisplayGoTerm?id=GO:0046933) |
| 20^C^ | 6.8/60.3 | 5.50/40.12 | 29.0% | 7(17) | 82 | Enolase (EC 4.2.1.11) | gi|110761968 | B | [phosphopyruvate hydratase activity](http://www.ebi.ac.uk/ego/DisplayGoTerm?id=GO:0004634) |
| 22^C^ | 8.1/37.9 | 6.26/36.06 | 32.0% | 12(65) | 105 | Aldehyde reductase | [gi|66525576](http://www.matrixscience.com/cgi/protein_view.pl?file=../data/20081222/FtTuliaEh.dat&hit=1) | B | [oxidoreductase activity](http://www.ebi.ac.uk/ego/DisplayGoTerm?id=GO:0016491) |
| 27^C^ | 8.4/72.1 | 7.6/67.41 | 21.0% | 15(39) | 90 | Transketolase (EC 2.2.1.1) | [gi|110751363](javascript:e('examine.asp','110751363','080925-143121-222.128.5.60-6083');) | B | [transketolase activity](http://www.ebi.ac.uk/ego/DisplayGoTerm?id=GO:0004802) |
| **Amino acid and fatty acid metabolism** | | | | | | | | | |
| 4^C^ | 5.9/16.1 | 5.50/15.55 | 57.0% | 6(29) | 83 | Fatty acid binding protein | gi|58585214 | A | [lipid binding](http://www.ebi.ac.uk/ego/DisplayGoTerm?id=GO:0008289)；[transporter activity](http://www.ebi.ac.uk/ego/DisplayGoTerm?id=GO:0005215) |
| 16^C^ | 3.0/26.1 | 4.83/26.82 | 14.0% | 8(39) | 126 | Proteasome subunit alpha type 5 (EC 3.4.25.1) | [gi|66541426](http://www.ncbi.nlm.nih.gov/blast/Blast.cgi?ALIGNMENTS=50&ALIGNMENT_VIEW=Pairwise&AUTO_FORMAT=Semiauto&CDD_SEARCH=on&CLIENT=web&COMPOSITION_BASED_STATISTICS=on&DATABASE=nr&DESCRIPTIONS=100&ENTREZ_QUERY=(none)&EXPECT=10&FILTER=L&FORMAT_BLOCK_ON_RESPAGE=None&FORMAT_OBJECT=Alignment&FORMAT_TYPE=HTML&GAPCOSTS=11+1&I_THRESH=0.001&LAYOUT=TwoWindows&MATRIX_NAME=BLOSUM62&NCBI_GI=on&PAGE=Proteins&PROGRAM=blastp&QUERY=MFLTRSEYDHGVNTFSPEGRLFQVEYAIEAIKLGSTAIGIATSEGVVLVVEKRITSSLMEPTTVEKIVEIDKHIGCAASGLIADSRTMIDRARVECQNHWFVYNERMSVESTAQAVSNLAIQFGDSDDDGSAMSRPFGVAMLFAGIDEKGPQLYHMDPSGTFVQFDAKAIGSGNEGAQQNLQEVYHKSMTLQEAIKAALVILKQVMEEKLSDNNIEVMTMTPEKLFHMFTKAELQEVIKDIA&SERVICE=plain&SET_DEFAULTS.x=9&SET_DEFAULTS.y=5&SHOW_OVERVIEW=on&WORD_SIZE=3&END_OF_HTTPGET=Yes) | B | [threonine-type endopeptidase activity](http://www.ebi.ac.uk/ego/DisplayGoTerm?id=GO:0004298) |
| 21^Q^ | 7.6/67.5 | 5.94/83.33 | 14.0% | 7(10) | 85 | long-chain-fatty-acid CoA ligase (EC [6.2.1.3](http://www.expasy.org/enzyme/6.2.1.3)) | gi|110762211 | B | [long-chain fatty acid-CoA ligase activity](http://www.ebi.ac.uk/ego/DisplayGoTerm?id=GO:0004467) |
| **Antioxidant system** | | | | | | | | | |
| 5^W^  8^W^  14^W^  18^Q^ | 6.7/18.2  6.6/17.6  8.2/45.4  4.7/18.4 | 5.65/21.78  5.65/21.78  5.65/21.94  5.65/21.94 | 32.0%  50.0%  12.0%  32.0% | 10(36)  8(19)  2(17)  6(9) | 90  117  141  99 | Thioredoxin peroxidase 1 (EC 1.11.1.15) | [gi|66548188](javascript:e('examine.asp','66548188','081022-122428-222.128.5.60-1428');) | A  A  A  B | [peroxidase activity](http://www.ebi.ac.uk/ego/DisplayGoTerm?id=GO:0004601) |
| 9^C^ | 7.2/15.9 | 5.88/25.12 | 23.0% | 5(9) | 81 | Peroxiredoxin 2540 (EC [1.11.1.7](http://www.expasy.org/enzyme/1.11.1.7)) | [gi|66535082](http://www.ncbi.nlm.nih.gov/blast/Blast.cgi?ALIGNMENTS=50&ALIGNMENT_VIEW=Pairwise&AUTO_FORMAT=Semiauto&CDD_SEARCH=on&CLIENT=web&COMPOSITION_BASED_STATISTICS=on&DATABASE=nr&DESCRIPTIONS=100&ENTREZ_QUERY=(none)&EXPECT=10&FILTER=L&FORMAT_BLOCK_ON_RESPAGE=None&FORMAT_OBJECT=Alignment&FORMAT_TYPE=HTML&GAPCOSTS=11+1&I_THRESH=0.001&LAYOUT=TwoWindows&MATRIX_NAME=BLOSUM62&NCBI_GI=on&PAGE=Proteins&PROGRAM=blastp&QUERY=MRINSIVPNFEADTTQGQINFYDWQGDSWVVLFSHPADFTPVCTTELGRLAVHQPHFKRRNTKLLAHSVDKLQDHVDWVNDIKSYCQDIPGAFPYPIIADHDRTLAVKLDMIDEISKDDPEQALTVRALYIISPDHRLRLSMHYPTSTGRNVDEILRVIDSLQLVDKRPEIATPANWVPGEKVMILPTVKDEELPKLFPKGVDKVSMPSGKIYVRTTTNY&SERVICE=plain&SET_DEFAULTS.x=9&SET_DEFAULTS.y=5&SHOW_OVERVIEW=on&WORD_SIZE=3&END_OF_HTTPGET=Yes) | A | [glutathione peroxidase activity](http://www.ebi.ac.uk/ego/DisplayGoTerm?id=GO:0004602); [thioredoxin peroxidase activity](http://www.ebi.ac.uk/ego/DisplayGoTerm?id=GO:0008379) |
| **Development** | | | | | | | | | |
| 2^W^ | 4.7/38.2 | 4.78/29.09 | 36.0% | 10(21) | 98 | 14-3-3 protein epsilon | [gi|48096523](http://www.ncbi.nlm.nih.gov/blast/Blast.cgi?ALIGNMENTS=50&ALIGNMENT_VIEW=Pairwise&AUTO_FORMAT=Semiauto&CDD_SEARCH=on&CLIENT=web&COMPOSITION_BASED_STATISTICS=on&DATABASE=nr&DESCRIPTIONS=100&ENTREZ_QUERY=(none)&EXPECT=10&FILTER=L&FORMAT_BLOCK_ON_RESPAGE=None&FORMAT_OBJECT=Alignment&FORMAT_TYPE=HTML&GAPCOSTS=11+1&I_THRESH=0.001&LAYOUT=TwoWindows&MATRIX_NAME=BLOSUM62&NCBI_GI=on&PAGE=Proteins&PROGRAM=blastp&QUERY=MSEREDNVYKAKLAEQAERYDEMVEAMKKVASLDVELTVEERNLLSVAYKNVIGARRASWRIISSIEQKEENKGAERKLEMIRQYRSQVEKELKDICADILGVLDKHLLPCASTGESKVFYYKMKGDYHRYLAEFAVGNDRKEAAENSLVAYKAASDTAMTDLPPTHPIRLGLALNFSVFYYEILNSPDRACRLAKAAFDDAIAELDTLSEESYKDSTLIMQLLRDNLTLWTSDMQGDGEGEQKEQLPDVEDQDVS&SERVICE=plain&SET_DEFAULTS.x=9&SET_DEFAULTS.y=5&SHOW_OVERVIEW=on&WORD_SIZE=3&END_OF_HTTPGET=Yes) | A | [protein domain specific binding](http://www.ebi.ac.uk/ego/DisplayGoTerm?id=GO:0019904) |
| 10^C^ | 7.2/35.2 | 6.50/30.01 | 52.2% | 9 (39) | 89 | Lethal(2)37 | gi|48097857 | A | [protein binding](http://www.ebi.ac.uk/ego/DisplayGoTerm?id=GO:0005515) |
| 24^C^  26^Q^ | 7.8/61.1  8.0/64.4 | 8.06/49.00  8.06/49.00 | 15.0%  20.0% | 7(47)  5(9) | 154  86 | Imaginal disc growth factor 4 | [gi|66514614](http://www.matrixscience.com/cgi/protein_view.pl?file=../data/20081127/FtTpmxcEE.dat&hit=1) | B  B | [imaginal disc growth factor activity](http://www.ebi.ac.uk/ego/DisplayGoTerm?id=GO:0008084) |
| **Protein folding** | | | | | | | | | |
| 7^W^  19^C^ | 6.2/76.9  5.5/69.7 | 5.20/72.48  5.4/71.77 | 18.4%  19.0% | 13(29)  12(46) | 122  109 | Heat shock protein 8 | [gi|66537940](javascript:e('examine.asp','66537940','080925-141203-222.128.5.60-4316');) | A  B | [unfolded protein binding](http://www.ebi.ac.uk/ego/DisplayGoTerm?id=GO:0051082) |
| **Transcription/translation** | | | | | | | | | |
| 15^W^  1^W^ | 8.4/62.4  4.6/39.1 | 4.57/19.83  4.57/19.83 | 11.0%  15.0% | 6(31)  6(27) | 141  147 | Translational controlled tumor protein | [gi|66515987](http://www.ncbi.nlm.nih.gov/blast/Blast.cgi?ALIGNMENTS=50&ALIGNMENT_VIEW=Pairwise&AUTO_FORMAT=Semiauto&CDD_SEARCH=on&CLIENT=web&COMPOSITION_BASED_STATISTICS=on&DATABASE=nr&DESCRIPTIONS=100&ENTREZ_QUERY=(none)&EXPECT=10&FILTER=L&FORMAT_BLOCK_ON_RESPAGE=None&FORMAT_OBJECT=Alignment&FORMAT_TYPE=HTML&GAPCOSTS=11+1&I_THRESH=0.001&LAYOUT=TwoWindows&MATRIX_NAME=BLOSUM62&NCBI_GI=on&PAGE=Proteins&PROGRAM=blastp&QUERY=MKIYKDIFTGDEMFSDTYKIKLVDDVLYEVYGKVITRKSGDIEIAGFNPSAEEADEGTDESVESGVDIVMNHRLQETFAFGDKKSYTLYLKDYMKKLVAKLEEQAPDQVEVFKKNTNKVMKDILSRFNDLQFFTGESMDIDGIVALLEYREIDDESVPVLMLFKHGLEEQKF&SERVICE=plain&SET_DEFAULTS.x=9&SET_DEFAULTS.y=5&SHOW_OVERVIEW=on&WORD_SIZE=3&END_OF_HTTPGET=Yes) | A  A | [guanyl-nucleotide exchange factor activity](http://www.ebi.ac.uk/ego/DisplayGoTerm?id=GO:0005085) |
| **Unknown** | | | | | | | | | |
| 17^Q^ | 4.50/16.1 | 8.93/29.662 | 32.0% | 5(7) | 86 | GE14749 | gi|195495050 | B |  |

All the identified proteins were hit against A. mellifera. Spot Number corresponds to the number of protein spots in Figure 2B, the superscript “Q”on the spot number indicate the spot is only in queen larvae, “W” indicate the spot is only in worker larvae, and “C” indicate the spot is expressed both in queen and worker larvae. Theoretical molecular weight (*M*r) and isoelectric point (p*I*) of the identified proteins were retrieved from the protein database of NCBInr. Experimental *M*r and p*I* were calculated with PDQuest Software and internal standard molecular mass markers. Sequence coverage is the ratio of the number of amino acids in every peptide that matches with the mass spectrum divided by the total number of amino acids in the protein sequence. Matched peptide is the number of paring an experimental fragmentation spectrum to a theoretical segment of protein and searched is the total searched peptide. Mascot score is search against the database NCBInr. Protein name is given when proteins were identified by MALDI-TOF/MS and LC-Chip/ESI-QTOF-MS. Accession number is the unique number given to mark the entry of a protein in the database NCBInr. The letter “A” in “Up/down Regulated” represent worker larvae upregulated and queen larvae down regulated, “B” represents worker larvae down regulated and queen larvae upregulated.
