# Supplementary material for: Differential Protein Expression in Honeybee (Apis mellifera L.) Larvae: Underlying Caste Differentiation
Source: PLoS One. 2010 Oct 20;5(10):e13455. doi: 10.1371/journal.pone.0013455 (PMC2958119; doi:10.1371/journal.pone.0013455)
Supplement: Table S4 — Differentially expressed proteins of honeybee worker and queen larvae at 120 hours. (0.10 MB DOC) [file pone.0013455.s004.doc]

**Table S4. Differentially expressed proteins of honeybee worker and queen larvae at 120 hours**

| Spot  Number | Experimental  p*I* /*M*r(kDa) | Theoretical  p*I* /*M*r(kDa) | Sequence Coverage | Matched/Searched | Score | Protein Name | Accession Number | Up/down Regulated | Molecular function |
| --- | --- | --- | --- | --- | --- | --- | --- | --- | --- |
| **Carbohydrate metabolism and energy production** | | | | | | | | | |
| 34^W^  45^C^ | 6.0/29.3  5.0/61.3 | 5.25/55.10  5.25/55.10 | 25.0%  47.0% | 10(19)  20(42) | 180  175 | ATP synthase beta subunit (EC 3.6.3.14) | gi|110762902 | A  B | [hydrogen ion transporting ATP synthase activity, rotational mechanism](http://www.ebi.ac.uk/ego/DisplayGoTerm?id=GO:0046933) |
| 58^Q^  59^Q^  36^C^ | 8.4/72.1  8.2/76.0  6.3/61.5 | 7.6/67.41  7.62/67.76  7.62/67.76 | 21.0%  23.0%  24.0% | 15(39)  8(16)  9(17) | 90  88  99 | Transketolase (EC 2.2.1.1) | [gi|110751363](javascript:e('examine.asp','110751363','080925-143121-222.128.5.60-6083');) | B  B  A | [transketolase activity](http://www.ebi.ac.uk/ego/DisplayGoTerm?id=GO:0004802) |
| 52^Q^  54^Q^ | 7.9/48.4  7.9/39.3 | 6.69/55.65  6.69/55.65 | 21.0%  22.0% | 9(33)  9(20) | 80  103 | Aldehyde dehydrogenase (EC 1.2.1.5) | [gi|66530423](http://www.ncbi.nlm.nih.gov/blast/Blast.cgi?ALIGNMENTS=50&ALIGNMENT_VIEW=Pairwise&AUTO_FORMAT=Semiauto&CDD_SEARCH=on&CLIENT=web&COMPOSITION_BASED_STATISTICS=on&DATABASE=nr&DESCRIPTIONS=100&ENTREZ_QUERY=(none)&EXPECT=10&FILTER=L&FORMAT_BLOCK_ON_RESPAGE=None&FORMAT_OBJECT=Alignment&FORMAT_TYPE=HTML&GAPCOSTS=11+1&I_THRESH=0.001&LAYOUT=TwoWindows&MATRIX_NAME=BLOSUM62&NCBI_GI=on&PAGE=Proteins&PROGRAM=blastp&QUERY=MLRLLKKVRLSRYFSTATRPEPERNPAILYTGIFIDNEWHRSKSGKTFPTINPTTGETIAEIQEGDDADIDLAVNAANKAFKLGSPWRTMDASQRGVLLNNLASLMERHRAYLAALETLDNGKPYSDAYEFDVPSSIATLRYYAGWADKNHGQVIPIDGKYLAYTRHEPVGVCGQIIPWNFPILMMAWKLGPALATGNVIVLKPAEQTSLTALYIAQLCKDAGFPPGVINVVPGFGKTGAALVAHNLVDKIAFTGSTEVGKLIKQGAAMSNLKRTTLELGGKSPNIILSDVNLDQAVEAAHFGLFYNMGQCCCAGSRTFVEDSIYDEFVERSAARAKSRVVGNPFDSNVEQGPQIDEEQVNKIMSMIESGKNEGAELVSGGTRIGDKGYFVAPTVFANVKDYMTIAKEEIFGPVQQILKFSSLNEVITRANNTDYGLAAAVFTKDIDKANYIIQGLRAGTVWVNAYNVLTPQVPFGGFKMSGHGRELGQYGLEAYTEVKSVIVKVNQKNS&SERVICE=plain&SET_DEFAULTS.x=9&SET_DEFAULTS.y=5&SHOW_OVERVIEW=on&WORD_SIZE=3&END_OF_HTTPGET=Yes) | B  B | [aldehyde dehydrogenase (NAD) activity](http://www.ebi.ac.uk/ego/DisplayGoTerm?id=GO:0004029) |
| 42^Q^ | 4.9/40.8 | 5.5/40.12 | 29.0% | 7(17） | 82 | Enolase (EC 4.2.1.11) | gi|110761968 | B | [phosphopyruvate hydratase activity](http://www.ebi.ac.uk/ego/DisplayGoTerm?id=GO:0004634) |
| 60^Q^ | 8.6/60.8 | 8.15/44.96 | 20.0% | 8(13) | 98 | Phosphoglycerate kinase (EC 2.7.2.3) | [gi|110763826](http://www.matrixscience.com/cgi/protein_view.pl?file=../data/20080925/FtTcrnuST.dat&hit=1) | B | [phosphoglycerate kinase activity](http://www.ebi.ac.uk/ego/DisplayGoTerm?id=GO:0004618) |
| 53^Q^ | 7.8/32.5 | 8.40/28.94 | 36.0%, | 10(59) | 98 | Phosphoglycerate mutase (EC 5.4.2.1) | gi|66550890 | B | [2,3-bisphosphoglycerate-dependent phosphoglycerate mutase activity](http://www.ebi.ac.uk/ego/DisplayGoTerm?id=GO:0046538) |
| **Amino acid and fatty acid metabolism** | | | | | | | | | |
| 51^Q^ | 7.6/68.9 | 5.94/83.33 | 17.0% | 12(20) | 85  129 | long-chain-fatty-acid CoA ligase (EC [6.2.1.3](http://www.expasy.org/enzyme/6.2.1.3)) | gi|110762211 | B | [long-chain fatty acid-CoA ligase activity](http://www.ebi.ac.uk/ego/DisplayGoTerm?id=GO:0004467) |
| 55^Q^ | 8.0/55.5 | 8.5/47.34 | 26.0% | 9(29) | 98 | Ornithine aminotransferase precursor (EC 2.6.1.13) | [gi|66524972](javascript:e('examine.asp','66524972','080925-145448-222.128.5.61-1203');) | B | [ornithine-oxo-acid transaminase activity](http://www.ebi.ac.uk/ego/DisplayGoTerm?id=GO:0004587) |
| 35^W^ | 6.2/52.3 | 5.66/39.98 | 41.6% | 18(74) | 90 | Arginine kinase (EC [2.7.3.3](http://www.expasy.org/enzyme/2.7.3.3)) | gi|58585146 | A | [arginine kinase activity](http://www.ebi.ac.uk/ego/DisplayGoTerm?id=GO:0004054) |
| 30^C^  33^C^ | 5.5/15.3  5.8/15.3 | 5.46/15.54  5.50/15.55 | 84.0%  57.00% | 11(17)  6(29) | 166  83 | Fatty acid binding protein | gi|58585214 | A  A | [lipid binding](http://www.ebi.ac.uk/ego/DisplayGoTerm?id=GO:0008289)；[transporter activity](http://www.ebi.ac.uk/ego/DisplayGoTerm?id=GO:0005215) |
| **Antioxidant system** | | | | | | | | | |
| 31^W^  47^C^ | 5.8/22.5  5.8/16.5 | 5.65/21.94  5.65/21.94 | 49.0%  38.0% | 6(12)  6(17) | 94  83 | Thioredoxin peroxidase 1 (EC 1.11.1.15) | [gi|66548188|](javascript:e('examine.asp','66548188','081022-122428-222.128.5.60-1428');) | A  B | [peroxidase activity](http://www.ebi.ac.uk/ego/DisplayGoTerm?id=GO:0004601) |
| 32^W^ | 5.9/30.5 | 5.24/27.63 | 37.0% | 10(22) | 93 | short-chain dehydrogenase/  reductase (EC [1.1.1](http://www.expasy.org/enzyme/1.1.1.-)) | gi|66560290 | A | [oxidoreductase activity](http://www.ebi.ac.uk/ego/DisplayGoTerm?id=GO:0016491) |
| 46^C^ | 5.8/29.7 | 5.40/17.68 | 48.0% | 7(29) | 85 | Glutathione S transferase S1 (EC [2.5.1.18](http://www.expasy.org/enzyme/2.5.1.18)) | gi|66534655 | B | [glutathione peroxidase activity](http://www.ebi.ac.uk/ego/DisplayGoTerm?id=GO:0004602) |
| **Nutrition Storage** | | | | | | | | | |
| 40^W^  41^W^  39^W^  38^W^  37^W^ | 7.0/67.7  7.3/68.6  6.9/69.3  6.7/67.9  6.6/84.7 | 6.4/113.96  6.72/79.48  6.43/111.92  6.4/113.96  6.43/112.17 | 14.0%  10.0%  14.0%  16.0%  20.0% | 12(30)  6(8)  11(28)  14(34)  14(40) | 110  82  84  90  91 | Larval serum protein 2 | [gi|110761029](javascript:e('examine.asp','110761029','081222-233333-222.128.5.61-8888');) | A  A  A  A  A | [nutrient reservoir activity](http://www.ebi.ac.uk/ego/DisplayGoTerm?id=GO:0045735) |
| 50^Q^  44^C^ | 7.1/71.6  4.5/65.8 | 6.72/79.535  6.72/79.48 | 18.0%  10.0% | 12(24)  6(8) | 98  82 | Larval serum protein 1 | [gi|58585148](http://www.matrixscience.com/cgi/protein_view.pl?file=../data/20081222/FtTuliaST.dat&hit=1) | B  B | [nutrient reservoir activity](http://www.ebi.ac.uk/ego/DisplayGoTerm?id=GO:0045735) |
| **Development** | | | | | | | | | |
| 29^W^ | 5.1/51.8 | 5.30/42.201 | 26.0% | 8(13) | 92 | Actin-87E isoform 2 | gi|66509769 | A | [ATP binding](http://www.ebi.ac.uk/ego/DisplayGoTerm?id=GO:0005524) |
| 28^W^ | 5.3/47.6 | 5.9/42.659 | 33.0% | 12(54) | 85 | Cathepsin D (EC [3.4.23.1](http://www.expasy.org/enzyme/3.4.23.1)) | gi|66560290 | A | [aspartic-type endopeptidase activity](http://www.ebi.ac.uk/ego/DisplayGoTerm?id=GO:0004190) |
| 56^C^ | 8.0/64.4 | 8.06/49.00 | 20.0% | 5(9) | 86 | Imaginal disc growth factor 4 | [gi|66514614](http://www.matrixscience.com/cgi/protein_view.pl?file=../data/20081127/FtTpmxcEE.dat&hit=1) | B | [imaginal disc growth factor activity](http://www.ebi.ac.uk/ego/DisplayGoTerm?id=GO:0008084) |
| 43^Q^ | 4.5/42.1 | 4.79/28.17 | 31.0% | 10(32) | 185 | Leonardo | [gi|48097086](http://www.matrixscience.com/cgi/protein_view.pl?file=../data/20081202/FtTplnaTS.dat&hit=1) | B | [protein domain specific binding](http://www.ebi.ac.uk/ego/DisplayGoTerm?id=GO:0019904) |
| **Protein folding** | | | | | | | | | |
| 49^Q^ | 6.2/76.9 | 5.20/72.48 | 18.4% | 13(29) | 122 | Heat shock protein 8 | [gi|66537940](javascript:e('examine.asp','66537940','080925-141203-222.128.5.60-4316');) | B | [unfolded protein binding](http://www.ebi.ac.uk/ego/DisplayGoTerm?id=GO:0051082) |
| 48^Q^ | 5.8/67.0 | 5.57/55.82 | 23.0% | 8(16) | 100 | ERp60 (EC [5.3.4.1](http://www.expasy.org/enzyme/5.3.4.1)) | [gi|66546657](http://www.matrixscience.com/cgi/protein_view.pl?file=../data/20081022/FtToinSST.dat&hit=1) | B | [protein disulfide isomerase activity](http://www.ebi.ac.uk/ego/DisplayGoTerm?id=GO:0003756) |
| **Transcription/translation** | | | | | | | | | |
| 57^Q^ | 8.6/66.3 | 4.57/19.83 | 11.0% | 6(31) | 141 | Translational controlled tumor protein | [gi|66515987](http://www.ncbi.nlm.nih.gov/blast/Blast.cgi?ALIGNMENTS=50&ALIGNMENT_VIEW=Pairwise&AUTO_FORMAT=Semiauto&CDD_SEARCH=on&CLIENT=web&COMPOSITION_BASED_STATISTICS=on&DATABASE=nr&DESCRIPTIONS=100&ENTREZ_QUERY=(none)&EXPECT=10&FILTER=L&FORMAT_BLOCK_ON_RESPAGE=None&FORMAT_OBJECT=Alignment&FORMAT_TYPE=HTML&GAPCOSTS=11+1&I_THRESH=0.001&LAYOUT=TwoWindows&MATRIX_NAME=BLOSUM62&NCBI_GI=on&PAGE=Proteins&PROGRAM=blastp&QUERY=MKIYKDIFTGDEMFSDTYKIKLVDDVLYEVYGKVITRKSGDIEIAGFNPSAEEADEGTDESVESGVDIVMNHRLQETFAFGDKKSYTLYLKDYMKKLVAKLEEQAPDQVEVFKKNTNKVMKDILSRFNDLQFFTGESMDIDGIVALLEYREIDDESVPVLMLFKHGLEEQKF&SERVICE=plain&SET_DEFAULTS.x=9&SET_DEFAULTS.y=5&SHOW_OVERVIEW=on&WORD_SIZE=3&END_OF_HTTPGET=Yes) | B | [guanyl-nucleotide exchange factor activity](http://www.ebi.ac.uk/ego/DisplayGoTerm?id=GO:0005085) |

All the identified proteins were hit against A. mellifera. Spot Number corresponds to the number of protein spots in Figure 2B, the superscript “Q”on the spots number indicate the spot is only in queen larvae, “W” indicate the spot is only in worker larvae, and “C” indicate the spot is expressed both in queen and worker larvae. Theoretical molecular weight (*M*r) and isoelectric point (p*I*) of the identified proteins were retrieved from the protein database of NCBInr. Experimental *M*r and p*I* were calculated with PDQuest Software and internal standard molecular mass markers. Sequence coverage is the ratio of the number of amino acids in every peptide that matches with the mass spectrum divided by the total number of amino acids in the protein sequence. Matched peptide is the number of paring an experimental fragmentation spectrum to a theoretical segment of protein and searched is the total searched peptide. Mascot score is search against the database NCBInr. Protein name is given when proteins were identified by MALDI-TOF/MS and LC-Chip/ESI-QTOF-MS. Accession number is the unique number given to mark the entry of a protein in the database NCBInr. The letter “A” in “Up/down Regulated” represent worker larvae upregulated and queen larvae down regulated, “B” represents worker larvae down regulated and queen larvae upregulated.
